# Dose Recommendation of Remimazolam Tosilate for General Anesthesia in Children and Adolescents: Synergistic Combination of PopPK and PBPK Approaches

**DOI:** 10.3390/pharmaceutics18030315

**Published:** 2026-03-01

**Authors:** Qiong-Yue Liang, Hui-Hui Hu, Nassim Djebli, Yuan-Yuan Huang, Hao Jiang

**Affiliations:** 1 Jiangsu Hengrui Pharmaceuticals Co., Ltd., Lianyungang 222006, China; qiongyue.liang.ql3@hengrui.com (Q.-Y.L.); huihui.hu.hh20@hengrui.com (H.-H.H.); nassim.djebli@luzsana.com (N.D.); yuanyuan.huang@hengrui.com (Y.-Y.H.); 2 Luzsana Biotechnology Europe AG, 4051 Basel, Switzerland

**Keywords:** remimazolam, pediatric, population pharmacokinetics, physiologically based pharmacokinetic

## Abstract

**Background:** Remimazolam tosilate is a novel, ultra-short-acting benzodiazepine. To address the unmet clinical need for safe and controllable general anesthetic options in children and adolescents, both top-down (i.e., population pharmacokinetics—PopPK) and bottom-up (i.e., physiologically based PK—PBPK) modeling approaches were combined to leverage their respective strengths for dose selection in children and adolescents aged 3–18 years. **Methods**: Pooled PK data from adult studies were used to develop and verify the adult PopPK and PBPK models. The PopPK model included allometric scaling to describe body weight effects, while the PBPK modeling incorporated the age-dependent physiological and metabolic ontogeny. Potential covariates and intrinsic factors influencing remimazolam exposure were assessed. Both models were then applied to simulate PK and derive exposure metrics in 3–18-year-old children and adolescents. The predictions from both approaches were used to support pediatric dose selection using an adult-matching exposure approach. **Results**: The PopPK and PBPK model simulations yielded consistent exposure predictions and converged on the same recommended dosing regimens for the pediatric population, providing mutual confirmation of model reliability. Both models indicated that the proposed regimens of remimazolam would achieve systemic exposures in children and adolescents (3–18 years) comparable to those in adults receiving an induction dose of 0.3 mg/kg followed by maintenance infusions of 1.0 or 3.0 mg/kg/h. Two pediatric dosing regimens were recommended: 1. Lower dose group: induction 0.2 mg/kg, initial maintenance 1.0 mg/kg/h, titratable as needed, with a maximum rate of 3.0 mg/kg/h (up to 4.0 mg/kg/h for individuals ≤ 30 kg). 2. Higher dose group: induction 0.3 mg/kg, initial maintenance 2.0 mg/kg/h, titratable as needed, with a maximum rate of 3.0 mg/kg/h (up to 4.0 mg/kg/h for individuals ≤ 30 kg). The model-informed dosing regimens have received regulatory approval from the Center for Drug Evaluation (CDE) in China and are currently being evaluated in an ongoing clinical trial. **Conclusions**: The integrated PopPK–PBPK approach supports evidence-based dosing recommendations of remimazolam for general anesthesia in children and adolescents aged 3–18 years and provides a reference for dose selection in future clinical studies.

## 1. Introduction

Remimazolam tosilate is a novel, short-acting benzodiazepine sedative–hypnotic that acts on γ-aminobutyric acid A (GABAa) receptors [1]. Currently, remimazolam can be used safely and effectively for induction and maintenance of general anesthesia, procedural and intensive care units sedation, with its satisfactory sedative and hypnotic effects demonstrated in several clinical trials [2,3]. Remimazolam was rapidly metabolized by carboxylesterase 1 (CES1) into an inactive, renally excreted metabolite (CNS7054) [4]. Given this organ-independent metabolic pathway of remimazolam via CES1,and the extensive characterization of CES1 expression in both children and adults [5,6], remimazolam was potentially ideal for children with immature metabolic systems. Thus, remimazolam exhibits rapid onset, short duration of action, and predictable recovery, offering excellent controllability and hemodynamic stability in pediatric anesthesia settings [7,8].

Despite these pharmacological advantages and a clear unmet clinical need for safer, short-acting anesthetic agents in children, remimazolam has not yet been approved for pediatric use, and its administration in this population remains off-label, substantially limiting anesthetic options for pediatric patients. As such, remimazolam was expected to play a key role in the management of sedation and anesthesia across both adult and pediatric populations. Identifying an optimal pediatric dosing regimen represents a critical step toward regulatory approval and clinical implementation. However, pediatric studies were inherently difficult to conduct and were accompanied by unique ethical and logistical challenges. Because pharmacokinetics (PKs) in children usually differ substantially from those in adults, particularly due to developmental changes in enzyme activity and organ maturation, exposure matching between adults and children was essential to ensure comparable systemic drug exposure.

Quantitative pharmacology models offer a rational and efficient approach to optimize pediatric dosing. Population PK (PopPK) is a “top-down” data-driven method that describes observed PK but has limited extrapolation capabilities as it is data-driven. In contrast, physiologically based PK (PBPK) is a “bottom-up” and mechanism-based method that can predict outcomes in novel scenarios, even though it still requires a model verification with clinical data to reduce the associated uncertainty. The integration of both modeling approaches represents a cutting-edge “learn-and-confirm” paradigm in model-informed drug development, increasingly recognized by regulatory agencies for pediatric dose selection [9,10,11,12]. Together, PopPK and PBPK form a “mechanism–data” loop: The estimated PopPK structure and/or parameters validate and sometimes adjust the underlying PBPK theory, and PBPK often provides a physiological explanation to the parsimonious PopPK structure. This synergy enhances model credibility, maximizes data value, allows for more precise extrapolation, and creates a self-improving system, providing a stronger basis for regulatory decisions.

Several PopPK models of remimazolam have been published [13,14,15,16,17], effectively describing its PK characteristics in adults. However, to date, no full PBPK model has been reported, and no study has combined PopPK and PBPK approaches to support the pediatric dose selection.

The present analysis aimed at evaluating the performance of the PopPK model that incorporates the well-established allometric scaling, on the one hand, and the PBPK model that considers the well-described organ maturation and ontogeny profiles of the physiological processes and metabolizing enzymes, on the other hand. Moreover, this analysis aimed to assess how those models, built and/or verified with adult data, can reasonably and accurately predict the PK of remimazolam in pediatric populations and how these two complementary model-based approaches could be synergistically integrated to provide a stronger rationale toward the support of dose selection for remimazolam in children and adolescents.

## 2. Materials and Methods

### 2.1. General Workflow

**Figure 1:**
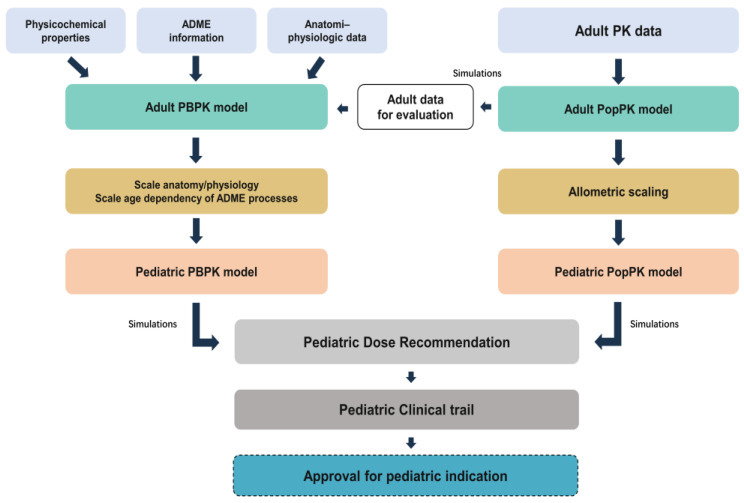
Workflow for developing the pediatric PBPK and PopPK models. PopPK, population pharmacokinetic model; PBPK, physiologically based pharmacokinetic model; ADME, adsorption, distribution, metabolism, and excretion.

The parallel development and qualification of PopPK and PBPK models of remimazolam in adults were conducted, as illustrated in the proposed workflow (Figure 1). First, a deep investigation was conducted to collect drug-specific physicochemical properties and absorption, distribution, metabolism, and elimination (ADME) characteristics. These parameters were used to build the adult PBPK model. Once adult PK data became available from two Phase I clinical studies, the PopPK model was built and then used for the PBPK model qualification, further increasing the model precision, as any deviation could be amplified during extrapolation to children. After the adult models were validated, both PBPK and PopPK models were extrapolated to the virtual pediatric population.

The extrapolation was based on three reasonable assumptions: (1) similarity of model structure in adults and children; (2) similarity of general anesthesia between adults and children and (3) the age-related variations were driven by organ maturation and ontogeny factors affecting physiology and metabolizing enzymes in the PBPK model, while only the body weight-driven theory-based allometric scaling and CES1 ontogeny were driving those changes in the PopPK model.

The PBPK model integrated drug-specific parameters (physicochemical properties, ADME data) with system-specific parameters (anatomical and physiological data such as cardiac output, tissue blood flow, tissue composition, etc.). After PBPK model verification and qualification, the virtual pediatric population, which incorporates physiological characteristics and ontogeny factors, was used to perform simulations in the pediatric individuals.

The PopPK model was extrapolated using theory-based allometric scaling to perform simulations in different age (or body weight) categories of virtual pediatric individuals, in the absence of any observed data in this population.

Once pediatric PK data were obtained, both models were optimized and qualified within the “learn–confirm paradigm” and followed a continuous improvement process.

### 2.2. Study Design and Study Population

The study datasets from two Chinese clinical trials (Study Numbers: HR7056-Ia and HR7056-Ib) were used for PBPK model refinement and qualification and for PopPK model building. Table 1 provides a summary of each study. In the current analysis, both arterial and venous blood concentration were used in the PBPK analysis, while the PopPK modeling was performed using arterial blood concentration data only. All trials were conducted in accordance with the Declaration of Helsinki and the International Conference on Harmonization Good Clinical Practice Guidelines. For each trial, the protocol was approved by the ethics committee at each center. All subjects provided written informed consent.

HR7056-Ia: A single-center, randomized, double-blind study evaluated single ascending doses of remimazolam administered as a 1 min intravenous (IV) injection, using midazolam as an active comparator. The trial comprised eleven dose groups (1–11) and enrolled 80 healthy Chinese male and female subjects.

HR7056-Ib: A single-center, randomized, double-blind, placebo-controlled, two-period crossover study included two groups. All subjects received a loading dose of 0.29 mg/kg remimazolam (IV infusion within 1 min) followed by a maintenance infusion of 1.08 mg/kg/h (over 2 h). At 1 h and 55 min after initiation of dosing, subjects received either a 0.5 mg IV bolus of flumazenil or an equal-volume saline placebo IV bolus. The study enrolled 8 healthy Chinese male and female subjects randomly allocated to two groups (*n* = 4 per group). Each participant underwent two dosing periods in this crossover design. Group 1 received flumazenil in Period 1 and saline placebo in Period 2. Group 2 received saline placebo in Period 1 and flumazenil in Period 2.

### 2.3. Blood Sample Collection and Analysis

In the Ia study, arterial and venous blood concentration samples of remimazolam were collected before and at 1, 2, 3, 4, 6, 8, 10, 12, 15, 20, 30, and 45 min, and 1, 2, 3, 4, 6, and 8 h post-dose after dosing. In the Ib study, arterial and venous blood was sampled before and at 1, 2, 3, 4, 6, 10, 15, 30, 60, 90, 115, 120 min after dosing, pre-infusion-cessation (119 min); moreover, blood samples were collected at 0, 1, 2, 3, 4, 6, 8, 10, 20, 40, 60, 90 min and 2.5, 4 h after dosing cessation.

All PK blood samples from arteries and veins were stored at −80 °C until analysis. Plasma concentrations of remimazolam were measured using ultra-performance liquid chromatography with tandem mass spectrometric detection, as described in our previous report [18]. The method was fully validated over the concentration range of 0.5–1000 ng/mL. The lower limit of quantification (LLOQ) was 0.5 ng/mL. Inter- and intra-batch precision and accuracy were well within the acceptable range. Selectivity, standard curve and quantitation range, sample stability, extraction recovery, and methodology quality control were also investigated.

### 2.4. Software

PopPK modeling was conducted using NONMEM (ICON Development Solutions, Hanover, MD, Version 7.5), running with Wings for NONMEM and PsN (perl-speaks-nonmem version 5.3.1, Uppsala, Sweden) serving as ancillary software for NONMEM execution.

The PBPK model was performed using PK-Sim (PK-Sim^®^, Version 12, part of Open System, Vienna, Austria, Vienna, Austria Pharmacology Suite, Bayer Technology Services, Leverkusen, Germany, http://www.open-systems-pharmacology.org/). Parameter optimization used the Levenberg–Marquardt algorithm in PK-Sim^®^.

Data integration was performed using R (version 4.0.3, The R Foundation for Statistical Computing, Vienna, Austria) and RStudio (version 2024.04.2, RStudio, Inc., Boston, MA, USA). Graphics and analytical results were compiled with R and RStudio.

### 2.5. PBPK Model Development and Evaluation

#### 2.5.1. Adult PBPK Model Development and Evaluation

To facilitate the application of remimazolam in the pediatric population, we initially developed the adult PBPK model. System-specific parameters (organ volumes, blood flow, hematocrit, etc.) were provided within the PK-Sim libraries. Drug-specific physicochemical parameters, such as molecular weight, lipophilicity (logP), fraction unbound in plasma and pKa, etc., were obtained from the literature. The values of logP and pKa were derived from the drugbank [19]. The plasma protein binding of remimazolam was approximately 92%, predominantly due to serum albumin [2]. PK-Sim offers five methods to compute the organ/plasma partition coefficient: Rodgers and Rowland (RR), PK-Sim standard, Poulin and Theil (PT), Schmitt and Berezhkovskiy, and three methods for cellular permeability: PK-Sim standard, charge-dependent Schmitt and charge-dependent Schmitt normalized to PK-Sim standard. The method resulting in the best fit of observed data was finally selected.

Remimazolam was rapidly metabolized by CES1 to an inactive carboxy acid metabolite (HR7054) [4]. CES1 was considered to be the only metabolic pathway in the model. To integrate enzyme-level drug metabolism with overall metabolism, PBPK models were enhanced by incorporating tissue- and age-specific protein expression data obtained from the Gene Expression Database on the Open Systems Pharmacology website. Three enzyme expression datasets were available: (1) whole genome expression arrays from Array Express (Array Express, 2010), (2) expressed sequence tags (ESTs) from UniGene and (3) RT-PCR (reverse transcription polymerase chain reaction)-derived gene expression estimates obtained from the literature. Based on the PK characteristics, the RT-PCR enzyme expression dataset was selected as this expression database allowed us to better capture the observed data. Based on the distinct enzymatic characteristics and metabolic behaviors of CES1, the first-order kinetics was used to describe CES1-mediated metabolism, and the CES1 clearance as well as organ permeability were optimized with the clinical observations. The PBPK model input parameters for remimazolam are reported in Table 2.

We set a virtual East Asian individual, aged 30, to represent the typical virtual patient within the population. To assess the model’s predictive accuracy, we conducted population simulations using a cohort of 100 virtual subjects, which were created to represent each clinical study based on their respective median data for age, weight, height, and body mass index. The observed data references, along with the dosing regimen and formulation details, are provided in Table 1. Clinical pharmacokinetic data were split into a training dataset for model development and optimization and an independent testing dataset for model evaluation.

The average fold error (AFE) was used to quantify the discrepancies between predicted and observed main PK parameter values (i.e., C max and AUC), thereby assessing the precision of the PBPK model predictions.

(1) $$\mathrm{AFE}=\frac{\mathrm{predicted}\;\mathrm{PK}\;\mathrm{parameter}}{\mathrm{observed}\;\mathrm{PK}\;\mathrm{parameter}}$$

We considered the model final if the mean ± SD of the simulated data captured >80% of the observed data within a 90% prediction interval and the predicted PK parameters should fall within a range of 0.5-to-2-times the corresponding observed value.

#### 2.5.2. Pediatric PBPK Model Extrapolation

Once the adult PBPK model was built and qualified, the model was applied on a virtual pediatric population by specifying the underlying age and weight. Pre-established age-dependent algorithms in PK-Sim^®^ were used to generate anatomical and physiological parameters, including body weight, height, organ weights and volumes, blood and lymph flows, cardiac output, total body water, lipid as well as lipid and protein concentrations, among other factors.

The metabolic processes of remimazolam were then scaled, and age-dependent CES1 protein developmental ontogeny, as represented by Equation (2), was added to the model [6], and CES enzymes were adjusted by enzyme abundance for a specific age. A comprehensive explanation of the enzymes and their maturation patterns with age is available in Supplementary Material Section S1 (Figure S1).

(2) $$\mathrm{FCES}1=\left(\frac{{\mathrm{Adult}}_{\max}-F_{\mathrm{birth}}}{{\mathrm{Age}50}^{n}+{\mathrm{Age}}^{n}}\right)\;\times \;{\mathrm{Age}}^{n}+F_{\mathrm{birth}}=\left(\frac{1-0.2}{1.10^{0.56}+{\mathrm{Age}}^{0.56}}\right)\times {\mathrm{Age}}^{0.56}+0.2$$

### 2.6. PopPK Model Development and Evaluation

#### 2.6.1. Base Model Development

The base model had a three-compartment structure to describe remimazolam concentrations over time [13]. The body weight was included using allometric scaling, similar to previously published remimazolam PopPK analyses in adults [16,21,22]. Accordingly, body weight was applied to all clearance (CL) and volume (V) parameters using the allometric scaling function in Equation (3) to enable physiologically plausible predictions in pediatric subjects:

(3) $$P_{\mathrm{TV}}\;=\;A\;\times {\;(\frac{\mathrm{Weight}}{{\mathrm{Weight}}_{median}})}^{b}$$

where P_TV_ represents the typical value of a PK parameter calculated for a subject with the median body weight; A denotes the typical parameter value at the median weight and b represents the allometric exponent (fixed to 0.75 for CL and intercompartmental clearances, Q2 and Q3, and 1.0 for V1 and volumes of the peripheral compartments, V2 and V3).

Inter-individual variability (IIV) was modeled using an exponential form (Equation (4)) for CL, V1, V2, V3, and Q3:

(4) $$P_{i}\;=\;P_{\mathrm{TV}}{\;\times \;e}^{\eta_{i}}$$

where P_i_ was the individual parameter estimate for subject i, P_TV_ was the typical population value, and η_i_ was inter-individual random effect for subject i, assumed to follow a normal distribution η_i_~N(0, ω^2^).

Residual error on arterial concentrations was modeled using an additive error model, as specified by Equation (5):

(5) $$C_{\mathrm{ij}}\;={\;C}_{\mathrm{TV},\;\mathrm{ij}}\;+\;\varepsilon_{a_{\mathrm{ij}}}$$

where C_ij_ was the jth observed drug concentration for subject i; C_TV,ij_ was the corresponding model-predicted drug concentration; and ε_aij_ was the additive residual error, which was assumed to follow a normal distribution: ε_aij_~N(0, σ^2^).

#### 2.6.2. Covariate Analysis

The Modification of Diet in Renal Disease (MDRD) equation [23] was used to calculate estimated glomerular filtration rate (eGFR, mL/min) (Equation (6)):

(6) $$eGFR=\frac{175\;\times \;{\left(\frac{\mathrm{CR}}{88.4}\right)}^{-1.234}\;\times \;{A\mathrm{ge}}^{-0.179}\;\times \;0.79(\mathrm{if}\;\mathrm{female})\;\times \;\mathrm{BSA}}{1.73}$$

where CR denotes serum creatinine (unit was μmol/L); and BSA denotes body surface area (unit was m^2^).

In addition, the derived covariates, fractional CES1 abundance in adults (FCES1) [6] and body mass index (BMI, kg/m^2^) were calculated using Equations (2) and (7), respectively.

(7) $$\mathrm{BMI}=\frac{\mathrm{Weight}}{{\left(\mathrm{Height}/100\right)}^{2}}$$

The assessed covariates for the PopPK model included sex, age, height, BMI, BSA, alanine aminotransferase (ALT), aspartate aminotransferase (AST), total bilirubin (TBIL), albumin (ALB), alkaline phosphatase (ALP), creatinine (CR), blood urea nitrogen (BUN), estimated glomerular filtration rate (eGFR), and FCES1. Specifically, FCES1 was assessed only on CL. Using the full dataset, only covariates with an absolute correlation coefficient |R| ≥ 0.3 were eligible for inclusion in subsequent covariate screening to characterize their effects on remimazolam plasma concentrations. When two or more covariates exhibited high correlation (R > 0.8; e.g., BSA~height/weight, AST~ALT, eGFR~CR/sex), the covariate producing the largest reduction in objective function value (OFV), steepest parameter–covariate relationship slope, and/or greater physiological/clinical relevance was retained in the full model, while other correlated covariates were excluded from further consideration.

A stepwise forward inclusion approach was followed, sequentially adding candidate covariates from prior screening steps while monitoring OFV changes. Covariates reducing OFV by >3.84 (χ^2^, *p* < 0.05) were retained, beginning with the most significant. This iterative process continued until no further significant reductions occurred. Subsequently, backward elimination refined the model. Covariates were removed sequentially; those causing an OFV increase >10.83 (χ^2^, *p* < 0.001) were retained due to significant contribution, while others were excluded permanently.

The decision to include a covariate was not based solely on the change in the OFV. In addition, goodness-of-fit plots (GOFs), the precision of estimates, biological plausibility, and the magnitude of IIV and residual variability were considered, with the goal to describe a conservative model with adequate precision for simulation.

The influence of continuous covariates on PK parameters was evaluated using the following linear or power function models (Equations (8)–(10)):

(8) $$P_{i}\;={\;P}_{\mathrm{TV}}\;\times \;\left(1+\theta_{x}\;\times \;\frac{{\mathrm{Cov}}_{i}}{{\mathrm{Cov}}_{m}}\right)$$

(9) $$P_{i}={\;P}_{\mathrm{TV}}\;\times \;{\left(\frac{{\mathrm{Cov}}_{i}}{{\mathrm{Cov}}_{m}}\right)}^{\theta_{x}}$$

(10) $$P_{i}=P_{\mathrm{TV}}\;\times \;e^{\theta_{x}\;\times \;\mathrm{COV}}$$

where Pi is the estimated parameter for subject i, and P_TV_ is the typical population value of the parameter. Cov_i_ is the covariate value for the subject i, Cov_m_ is the median (or reference) value of the covariate, and θ_x_ denotes the estimated coefficient quantifying the covariate’s effect on the parameter.

For categorical covariates, parameter changes were evaluated using the following model structures (Equations (11)–(13)):

(11) $$\theta_{i}=\theta_{\mathrm{TV}}+\theta_{x,\mathrm{cov}=\mathrm{xl}}\;\mathrm{if}\;\mathrm{cov}=x_{l}\;\mathrm{and}\;l\;\text{∈}\left(0,m-1\right)$$

(12) $$\theta_{i}=\theta_{\mathrm{TV}}\;\mathrm{if}\;\mathrm{cov}=X_{0}$$

(13) $$P_{i}=P_{\mathrm{TV}}+\theta_{x}\;\times \;\mathrm{Factor}$$

where θ_x,cov=xl_ denotes the change in the parameter relative to the reference level (0) when the covariate X was at level l, and the covariate X_0_ encompasses m levels, ranging from 0 to m−1. For binary covariates, Factor represents an indicator variable coded as 0 or 1, and Equation (13) was used to estimate the corresponding effect on the parameter.

#### 2.6.3. Model Performance

After model development, model performance was assessed using goodness-of-fit plots, prediction-corrected visual predictive checks (pcVPCs) and nonparametric bootstrap. GOFs were used to examine predicted values and residuals to assess potential bias in the model. For pcVPCs, simulations (*n* = 1000) of the final model and parameters were conducted and the observed data were compared with the model-predicted median, 5th, and 95th percentiles, along with their corresponding 95% prediction intervals over time. Nonparametric bootstrap was also conducted (*n* = 1000). Each bootstrapped dataset was fitted to the final model and the median and 95% confidence intervals of parameter estimates were calculated.

#### 2.6.4. Pediatric PopPK Model Extrapolation

After the final adult PopPK model was established and qualified, it was extrapolated to pediatric subjects using Monte Carlo simulation. The structural model, allometric scaling functions, and estimated inter-individual variability were retained, and individual pharmacokinetic parameters for virtual subjects were generated accordingly. Concentration–time profiles under candidate dosing regimens were then simulated, and the resulting pharmacokinetic metrics were subsequently calculated to support dose selection.

### 2.7. Pediatric Dose Recommendation Strategy

#### 2.7.1. Adult Target Exposure Range

To characterize the target exposure range for dose extrapolation, simulations were first conducted in adults using the final PopPK model of remimazolam. A virtual adult population (*n*= 1000) was generated with a body weight distribution following a normal distribution (mean = 63.4 kg, SD = 7.3 kg), consistent with the Phase Ia/Ib study demographic characteristics. Monte Carlo simulations were performed in NONMEM to predict plasma concentration–time profiles following the recommended general anesthesia dosing regimen: an induction bolus of 0.3 mg/kg administered over 1 min, followed by continuous maintenance infusions at 1.0 mg/kg/h and 3.0 mg/kg/h for 2 h. The simulated pharmacokinetic parameters (C_max_, C_2h_, and AUC_0–∞_) were used to define the adult target exposure range, expressed as the 90% confidence interval of predicted values.

#### 2.7.2. Pediatric Exposure Prediction and Dose Selection

A virtual East Asian pediatric population (*n* = 100, 50% female) covering ages from neonates to 18 years was generated using PK-Sim^®^ software (Version 12). The virtual subjects were divided into six age-based cohorts: Cohort 1, aged 12–<18 years; Cohort 2, aged 9–<12 years; Cohort 3, aged 6–<9 years; Cohort 4, aged 3–<6 years; Cohort 5, aged 1–<3 years; and Cohort 6, aged 0–<1 year. The demographic characteristics, including body weight, height, age, and BMI, were randomly generated according to the prespecified age.

The virtual pediatric population was subsequently used in both PopPK and PBPK models to simulate plasma concentration–time profiles under several intravenous dosing regimens. Simulations aimed to identify pediatric dosing schemes that result in exposure levels (C_max_, C_2h_, and AUC_0–∞_) that are comparable to the predefined adult target exposure range. Comparative analyses were then conducted between the exposure profiles derived from the PopPK and PBPK simulation approaches.

## 3. Results

### 3.1. Analysis Populations and Demographics

The HR7056-Ia and HR7056-Ib studies were included in this analysis, with a total of 71 healthy Chinese subjects and 1439 arterial and 518 venous PK samples. Baseline characteristics are summarized in Table S1. The dataset consisted of 14 females (19.7%) and 57 males (80.3%). The mean age was 28.6 years (range: 18–51 years), and the median baseline body weight was 62.8 kg (range: 50.2–83.8 kg).

The arterial and venous semi-log concentration–time profiles were characterized for each dose group. The pharmacokinetic data suggested rapid elimination kinetics, with near-complete clearance within 4 h post-dose, consistent with a typical three-compartment model. Notably, significant arterio-venous concentration gradients were observed. Venous concentrations increased rapidly but reached peak levels substantially lower than the corresponding arterial C_max_ values. During the elimination phase, venous concentrations remained slightly higher than contemporaneous arterial measurements, suggesting potential differences in drug distribution between vascular compartments (Figure S2).

### 3.2. PBPK Model

**Figure 2:**
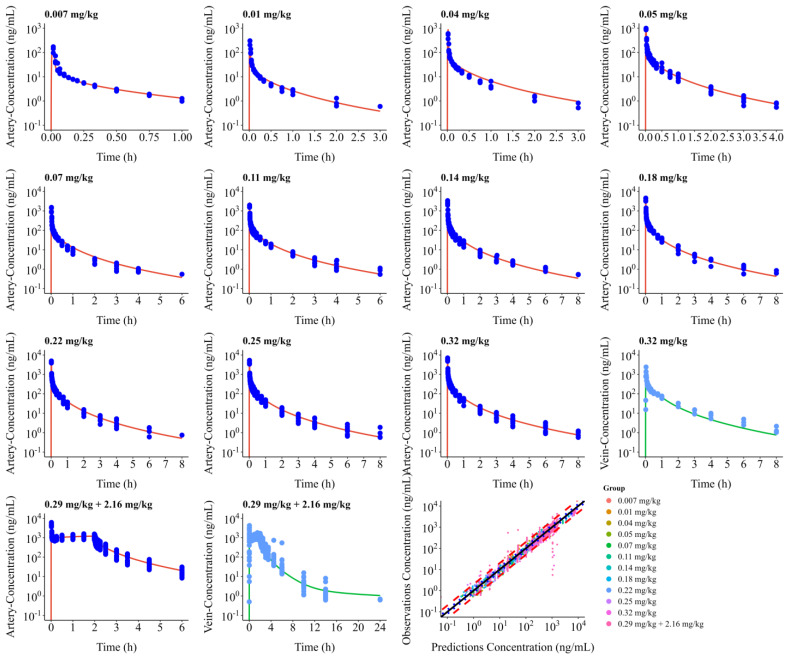
Concentration–time profiles of the final adult PBPK model—by dosing regimen. The last plot shows observed versus predicted concentration plot across dosing regimens.

We developed and validated a comprehensive PBPK model for remimazolam in adults, for both single intravenous dosing regimens across a wide dose range (0.007–0.32 mg/kg) and infusion dosing of 1.08 mg/kg/h. Tissue–plasma partition coefficients were estimated using the PK-Sim standard, while cellular permeabilities were determined using the PK-Sim standard. When we compared our optimized model with clinical data, the model met our prespecified acceptance criteria, demonstrating robust predictive performance. As shown in Figure 2, the model accurately captured the plasma concentration–time profiles across all dosing scenarios.

The model’s predictive accuracy was quantitatively assessed through comparison of key pharmacokinetic parameters (C_max_ and AUC_0–∞_) between simulated and observed data, as detailed in Table 3. And predicted and observed T_max_ values are summarized in Table S2. The average fold errors (AFEs) of the predicted/observed pharmacokinetic parameters C_max_ and AUC_0–∞_ were within the range of 0.82–1.75, and 0.88–1.51, respectively. All the AFEs values were within the range of 0.5–2.0. For dosing regimens of 0.29 and 0.32 mg/kg, the model tended to overestimate maximum concentrations (C_max)_ of the venous concentration–time profile. This might be due to the fast variation in the venous concentration within 10 min after administration. The predictive performance was further validated through GOF assessment, as shown in Figure 2. The plot revealed that 94% of predicted plasma concentrations fell within a 2-fold error range of observed values, confirming the reliability of the PBPK model.

### 3.3. PopPK Model

For remimazolam, a three-compartment model with linear clearance and first-order conditional estimation (OFV = −3632.983) could provide the best fit to the data, compared with a one-compartment model (OFV = 1348.978) or a two-compartment model (OFV = −949.542) with liner clearance. The first-order conditional estimation with Interaction (FOCEI) was selected for PopPK modeling in NONMEM.

The body weight was incorporated using allometric scaling. None of the other tested covariates met the predefined statistical criteria for inclusion in the final model based on the stepwise covariate modeling procedure. The results of the forward and backward stepwise procedures are detailed in Table S3.

The PopPK parameters from the final model are summarized in Table 4. The IIV and associated covariance were estimated with good precision, except for the IIV on the central volume of distribution (V1) that has a large relative standard error (RSE = 99.5%) for a very small IIV value (ω^2^ = 0.00546). This suggested that IIV on V1 was negligible and difficult to estimate precisely rather than reflecting any model instability. Removing this term resulted in no change in the objective function value (OFV) or diagnostic plots, supporting the interpretation that this term was negligible and does not affect model fit or predictive performance. Considering the correlation between ηCL and ηV1 and the improvement in OFV, an OMEGA block structure between CL and V1 was retained as the most appropriate random-effects model.

The GOF demonstrated that the PK model effectively described the observed concentrations (Figure S3). Bootstrap replicates showed similar median parameter estimates and comparable 95% CIs compared with the original NONMEM results (Table 4), further confirming model stability and robustness as well as the very low value of V1 of 0.0085 L with a 95% CI of [0.00164–0.0183]. As shown in Figure S4, the pcVPC demonstrated that the 95% prediction intervals of the median, 5th, and 95th percentiles of simulated concentrations appropriately captured the corresponding observed percentiles, with no apparent bias. Overall, the observed remimazolam concentration variability was well-captured by the estimated IIV. The final NONMEM control stream is provided in Supplementary Material Section S2.

### 3.4. Pediatric Dose Recommendation

#### 3.4.1. Target Exposure Range

Based on the final PopPK model, a simulated dataset of adult body weights was generated following a normal distribution (mean = 63.4 kg and SD = 7.3 kg) derived from the adult study population. A Monte Carlo simulation was then conducted to predict adult exposure under the recommended dosing regimen of remimazolam for injection. The simulated scenarios included: a bolus dose of 0.3 mg/kg administered over 1 min (induction phase); continuous infusion at 1.0 mg/kg/h or 3.0 mg/kg/h for 2 h (maintenance phase). The simulated concentration–time profile of remimazolam under the adult general anesthesia regimen is presented in Figure S5, and the corresponding exposure parameters (C_max_, C_2h_, and AUC_0–∞_) are summarized in Table 5.

#### 3.4.2. Pediatric PopPK Model Simulation

Using the developed PopPK model and virtual pediatric population, pediatric PK simulations were performed by applying allometric scaling for body weight. The virtual subjects were divided into seven age cohorts (<1, 1–3, 3–6, 6–9, 9–12, 12–18 and >18 years), corresponding to median body weights ranging from 6.6 to 57.0 kg (Table S2). For each cohort, same weight-adjusted dosing to adults (0.3 mg/kg induction + 1.0 mg/kg/h maintenance) was simulated.

**Figure 3:**
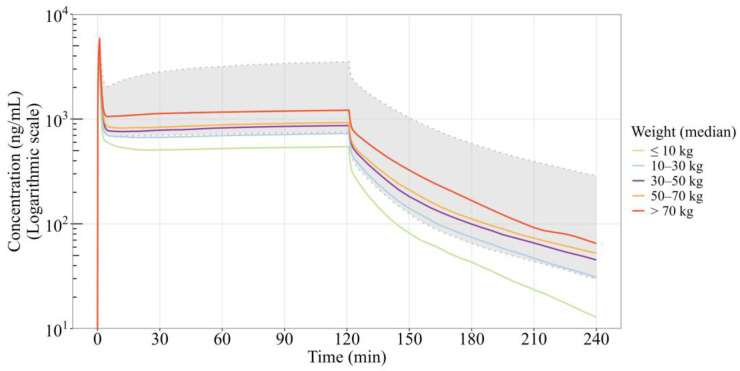
PopPK-simulated median plasma concentration–time profiles of remimazolam by body weight groups. The gray dashed area indicates the adult target exposure range (5th percentile of 0.3 mg/kg + 1.0 mg/kg/h and 95th percentile of 0.3 mg/kg + 3.0 mg/kg/h). Colored lines represent median concentrations for virtual pediatric subjects (0.3 mg/kg + 1.0 mg/kg/h).

Predicted drug concentrations increased slightly with body weight. Concentration–time profiles grouped by weight (≤10, 10–30, 30–50, 50–70, >70 kg) showed that the 30–50 kg and 50–70 kg groups had similar exposure levels and were comparable to those in adults, while subjects ≤30 kg exhibited lower exposure as compared to adults’ exposure range (gray dashed area, Figure 3).

#### 3.4.3. Pediatric PBPK Model Simulation

Based on the developed PBPK model, which incorporated physiological parameters and age-dependent CES1 enzyme ontogeny, simulations were executed for the pediatric population using PK-Sim^®^. The same seven virtual age cohorts (Table S5) were used for the simulations. The virtual pediatric population was administered an induction dose of 0.3 mg/kg bolus followed by a maintenance dose of 1.0 mg/kg/h continuous infusion. The PBPK-predicted exposure was then compared to that simulated by the PopPK model under the same dosing regimen. Consistent with PopPK predictions, drug exposure showed a slight increase with body weight or age. In particular, subjects aged <9 years exhibited lower exposure relative to children and adolescents with higher body weights. This age-based stratification aligns with the PopPK analysis, which identified a body weight of ~30 kg as a critical threshold; below this threshold, lower body weight was associated with reduced drug exposure.

**Figure 4:**
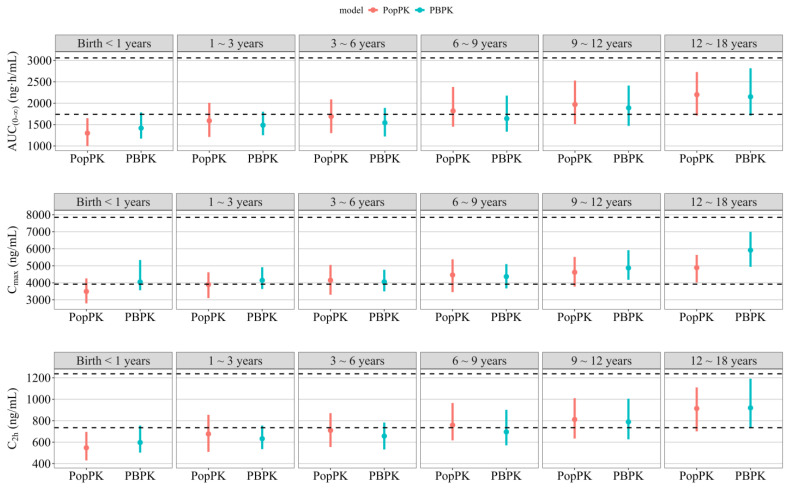
Comparison of PopPK with corresponding PBPK simulations by age groups at the same dose (0.3 mg/kg + 1.0 mg/kg/h). The two black dashed lines represent the upper and lower bounds of the adult exposure range, which is defined by the union of the 90% confidence intervals derived from simulations of both the PopPK and PBPK models. The points represent the median values, and the lines represent the 90% prediction interval.

The PBPK-predicted exposure parameters were comparable to those from PopPK simulations, with the ratios of median C_max_, C_2h_, and AUC_0–∞_ values within 1.25-fold across age groups (Figure 4, Table 6). This agreement represents a cross-validation of both modeling approaches for pediatric extrapolation.

#### 3.4.4. Final Dose Recommendation

**Figure 5:**
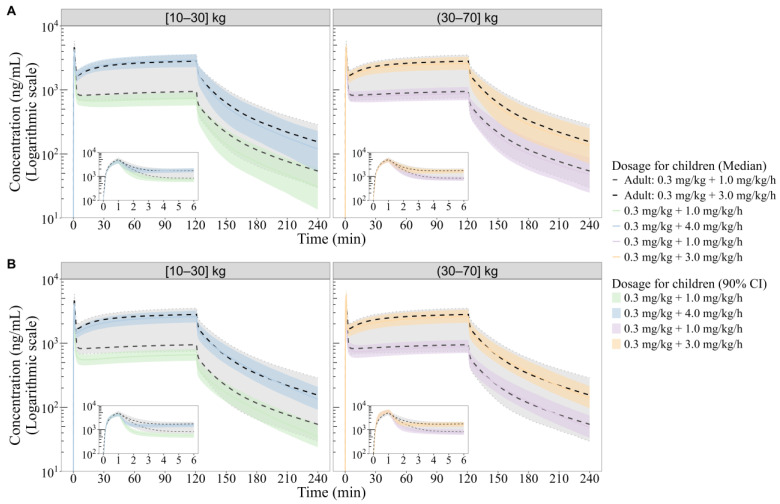
Simulated median plasma concentration–time profiles of remimazolam under different dosing regimens in virtual populations stratified by body weight using the PopPK model (**A**) and PBPK model (**B**). The gray dashed area indicates the adult target exposure range (5th percentile of 0.3 mg/kg + 1.0 mg/kg/h and 95th percentile of 0.3 mg/kg + 3.0 mg/kg/h regimens). Colored lines and shaded areas represent median and 5th–95th percentile ranges for maintenance doses of 1.0 mg/kg/h and 3.0 mg/kg/h. Green/blue (10–30 kg) and purple/orange (30–70 kg) lines and shaded curves represent median and 5th–95th percentile profiles for different dosing regimens.

Integrating PopPK and PBPK simulation results, the pediatric population was stratified into two weight-based dosing groups: 10–30 kg and 30–70 kg. For individuals >30 kg, the adult dosing regimen (induction: 0.3 mg/kg; maintenance: 1.0–3.0 mg/kg/h) provides exposures consistent with adult targets, requiring no adjustment. For individuals ≤30 kg, the same induction dose (0.3 mg/kg) can be maintained, while the upper maintenance infusion limit may be increased up to 4.0 mg/kg/h to achieve adult-matching exposures (Figure 5).

Simulations verifying these recommendations were conducted in two virtual pediatric populations (*n* = 1000 each) representing 10–30 kg and 30–70 kg years. Demographic details are summarized in Table S5, and simulated PK parameters are presented in Table 7.

## 4. Discussion

Dose optimization in pediatric populations remains a critical step in drug development, and the distinct ontogeny of pediatric physiology and pathology, together with profound age-dependent pharmacokinetic variability, imposes challenges on the rationale for guiding the dose selection. The efficacy and safety of remimazolam for the induction and maintenance of general anesthesia in children have been thoroughly described [24,25]. Compared to propofol, remimazolam has been associated with a lower incidence of hypotension and emergence delirium and a faster awakening during general anesthesia in pediatric patients. However, the data for regulatory submission that characterize the safety, efficacy and population PK/PD behavior of remimazolam during pediatric general anesthesia was lacking. Consequently, the optimal dosing regimens for both the induction and maintenance of anesthesia in pediatric patients was an unmet need.

Although previous studies enrolling children suggested that remimazolam exhibited PK profiles similar to adults [21]—supporting its potential for pediatric extrapolation—a model-informed drug development (MIDD) approach provided a powerful quantitative framework for rational dose selection. Traditional population pharmacokinetic (i.e., PopPK) modeling typically uses empirical methods such as allometric scaling to extrapolate adult data to children, yet struggles to account for ontogeny factors and organ maturation. In contrast, PBPK models offer a complementary approach for the precise prediction of pediatric doses by integrating age-related anatomical and physiological parameters. Several studies have successfully combined the two modeling methods [9,10,11,12]. This study synergistically integrated PopPK and PBPK modeling and simulation techniques to provide, to our knowledge, the first scientific basis for initial remimazolam dosing in pediatric patients.

Published PopPK models describing remimazolam pharmacokinetics have predominantly adopted two- or three-compartment structures with linear clearance [13,16,26,27]. Consistent with these reports, a three-compartment model with linear clearance was selected as the final structural model in the present analysis. With respect to covariate effects, published PopPK studies on remimazolam have shown inconsistent findings regarding body weight effects. For instance, Zhou et al. [13] observed no significant influence of weight on PK parameters, and Wiltshire’s [15] team reported similar findings. Gao et al. [21], in a pediatric population, also found no significant weight effect but applied allometric scaling to account for the specific characteristics of children, consistent with other PopPK reports on remimazolam that also applied the weight-based allometric models [16,22,28]. Considering these inconsistencies, in our covariate analysis, the inclusion of allometric scaling in our model reduced the objective function value (ΔOFV = 31.557) without compromising model precision. Considering the pediatric population, incorporating weight-based allometric scaling was therefore adopted as a conservative and more physiological strategy for extrapolating the adult model to pediatric populations. The estimated clearance and distribution parameters in the present model were within the ranges reported in previously published PopPK models, and the low relative standard errors together with limited shrinkage indicate robust parameter estimation.

Remimazolam was deactivated by carboxylesterase 1 (CES1) in the human liver, and the age-dependent absolute abundance of the CES1 enzyme affects drug metabolism. To our knowledge, CES ontogeny has mostly been investigated in vitro [5,6,29,30]. Boberg et al. [6] determined the absolute protein abundance of CES using liquid chromatography–tandem mass spectrometry (LC-MS/MS) proteomics. Their findings indicated that, in both hepatic microsomes and liver cytosol, CES1 protein abundance exhibited age-dependent maturation. Subsequently, the ontogeny data from birth were incorporated into the PBPK model to predict in vivo PK of remimazolam in pediatric populations. Incorporating the ontogeny of metabolic enzymes and pediatric physiological characteristics into PBPK models yields more reliable exposure predictions, particularly for infants and toddlers, as enzyme activity and organ function do not approach near-adult levels until approximately 2 years of age [9]. Consequently, we simulated the entire pediatric population, including those under three years old, to extend the potential benefits to a wider group of children.

Although remimazolam was metabolized by CES1 and CES1 immaturity was an important determinant of metabolic variability in very young children, published ontogeny data showed that CES1 activity increased rapidly after birth and reached near-adult levels by approximately three years of age, with only modest changes thereafter [5]. Considering this biological mechanism, incorporating the CES1 maturation function (FCES1) into the adult PopPK model resulted in only a negligible change in OFV and did not improve model diagnostics. Because the PopPK model was developed using adult data, where CES1 is fully mature, the limited influence of FCES1 on model performance was expected. When extrapolating this model to simulate children and adolescents aged 3–18 years—an age range in which CES1 activity is also close to adult levels—the impact of CES1 ontogeny on remimazolam clearance was therefore considered minimal. For these reasons, FCES1 was evaluated but not retained in the final PopPK model.

Based on the assumption that remimazolam’s mechanism of action was similar in adults and children, we set the therapeutic target exposure for pediatrics to be equivalent to the exposure range (and associated 90% CI) at the effective adult dose. By simulating various dosing regimens, we identified a recommended dose that would result in adult-matching exposures. The simulation results showed that the recommended pediatric dosing regimen, derived from model extrapolation, successfully achieved predicted pharmacokinetic parameters within the adult target exposure range. More importantly, the predictions from the PopPK and PBPK models demonstrated good consistency, mutually confirming the reliability of the extrapolation via a cross-validation of both approaches.

Finally, based on the model-informed approach, the recommended dosing regimens for general anesthesia in children and adolescents were as follows:

- Lower-dose group: induction 0.2 mg/kg; initial maintenance 1.0 mg/kg/h (titrated as needed); maximum 3.0 mg/kg/h (up to 4.0 mg/kg/h for individuals ≤ 30 kg).
- Higher-dose group: induction 0.3 mg/kg; initial maintenance 2.0 mg/kg/h (titrated as needed); maximum 3.0 mg/kg/h (up to 4.0 mg/kg/h for individuals ≤ 30 kg).

These recommended dosing regimens were simultaneously supported by findings from previously completed adult clinical studies of remimazolam. In registration trials in adults undergoing general anesthesia the actual induction doses administered ranged from 0.1 to 0.3 mg/kg, and the mean maintenance infusion rates achieved in clinical practice were 1.7 mg/kg/h and 2.6 mg/kg/h, respectively. The anesthesia induction success rates were 88.9–100%, and the safety profile was favorable. It was also supported by post-marketing pediatric experience with remimazolam in China [25,31], where an investigator-initiated study in 187 children aged 3–6 years reported a 100% success rate for anesthesia induction and a 99.3% success rate for anesthesia maintenance when using an induction dose of 0.3 mg/kg and a maintenance infusion rate of 2.0 mg/kg/h. The regimen was well-tolerated with a favorable safety profile. The lower induction dose of 0.2 mg/kg was proposed as a conservative option to minimize the risk of overexposure, while still allowing additional bolus dosing during induction if clinically required. Finally, the model-informed pediatric dose recommendation had been incorporated into the pediatric investigation plan for HR7056-207 (a pharmacokinetic, safety, and efficacy study of remimazolam tosilate for injection in children and adolescents undergoing general anesthesia in China; clinical trial registration No. CTR20252370). The predictive performance of the models will be further verified in this ongoing clinical trial.

Some limitations of the present analysis should be acknowledged. Both the PopPK and PBPK models were primarily developed using pharmacokinetic data from adult subjects; therefore, extrapolation to pediatric populations involves inherent uncertainty, which will require the incorporation of pediatric clinical data for external validation and further refinement. In addition, the dose recommendation was based on an adult-matching exposure approach. However, this approach comes with the assumption of similarity of exposure-response from both safety and efficacy perspectives.

Furthermore, the predictive reliability of pediatric PBPK models depends heavily on the accuracy of enzyme ontogeny functions and physiological parameterization; limitations in these aspects may affect model performance. In our work, although the simulations covered the entire pediatric age range of 0–18 years, the current clinical trial (HR7056-207) was designed to enroll children and adolescents aged from 3 to 18 years. This reflects a development strategy in which the initial evaluation focuses on older children, while younger children (<3 years) will be considered in future development stages if supportive data become available, consistent with a development approach that progresses from older to younger age groups. This decision was made to ensure confidence in dose selection in the absence of pediatric data, while also facilitating regulatory approval for pediatric and adolescent indications and addressing urgent clinical needs. Ongoing and future pediatric studies will be essential to validate model predictions and refine dosing recommendations based on observed pharmacokinetic, efficacy, and safety data.

## 5. Conclusions

Population and physiologically based pharmacokinetic models of remimazolam were developed using adult clinical data and extrapolated to pediatric populations. The two complementary modeling approaches adequately described the pharmacokinetics of remimazolam, providing consistent exposure predictions and converging on the same recommended dosing regimen. The integrated model-informed approach has received regulatory approval and supported dosing recommendations for children and adolescents in the ongoing trial.

## Figures and Tables

**Table 1:** Overview of the clinical studies used in the PopPK/PBPK analyses.

Study	Dose Regimen	PK Sampling Design	Subject Population	Planned Analytical Samples
Ia	Group1 (3A + 0P): 0.007 mg/kg, IV injection for 1 min	Arterial and venous blood samples were collected at 19 time points: pre-dose, and at 1, 2, 3, 4, 6, 8, 10, 12, 15, 20, 30, and 45 min, and 1, 2, 3, 4, 6, and 8 h post-dose.	Chinese adult healthy subjects	63 subjects (1197 samples)
	Group2 (3A + 0P): 0.01 mg/kg, IV injection for 1 min			
	Group3 (3A + 0P): 0.04 mg/kg, IV injection for 1 min			
	Group4 (6A + 1P): 0.05 mg/kg, IV injection for 1 min			
	Group5 (6A + 1P): 0.07 mg/kg, IV injection for 1 min			
	Group6 (6A + 2P): 0.11 mg/kg, IV injection for 1 min			
	Group7 (6A + 2P): 0.14 mg/kg, IV injection for 1 min			
	Group8 (6A + 2P): 0.18 mg/kg, IV injection for 1 min			
	Group9 (6A + 2P): 0.22 mg/kg, IV injection for 1 min			
	Group10 (9A + 3P): 0.25 mg/kg, IV injection for 1 min			
	Group11 (9A + 3P): 0.32 mg/kg, IV injection for 1 min			
Ib	In each dosing cycle, subjects received a loading dose of 0.29 mg/kg IV infusion within 1 min, while simultaneously receiving a maintenance dose of 1.08 mg/kg/h for 2 h. At 1 h and 55 min after dosing initiation, they received an intravenous bolus of either 0.5 mg flumazenil or an equal volume of saline. One group of subjects received flumazenil injection in the first cycle and saline injection in the second cycle; another group received saline injection in the first cycle and flumazenil injection in the second cycle.	Arterial and venous blood samples at 26 time points were obtained: pre-bolus; post-bolus (1, 2, 3, 4, 6, 10, 15, 30, 60, 90, 120 min); pre-infusion-cessation (119 min); and after dose-cessation (0, 1, 2, 3, 4, 6, 8, 10, 20, 40, 60, 90 min; 2.5, 4 h).	Chinese adult healthy subjects	8 subjects (416 samples)

Note: The dose of remimazolam is expressed as the free base.

**Table 2:** Drug-specific parameters used in PBPK model building.

Parameter	Values Used in the Model	References
Physicochemical parameters		
Molecular weight (g/mol)	439.31	[19]
Compound type	strong base	[19]
LogP	3.68	[19]
pKa	5.99	[19]
Plasma protein	albumin	[2]
Fraction unbound	0.08	[2]
Distribution		
Partition coefficient model	diverse	PK-Sim standard
Cellular permeability	diverse	PK-Sim standard
Organ-specific permeability (cm/min)	4.01 × 10^−4^ cm/min	Optimized
Metabolism		
First-order clearance by CES1		
Specific clearance (1/min)	50.37 1/min	[20]

LogP, lipophilicity; pKa, acid dissociation constant.

**Table 3:** PBPK-predicted and observed values for derived exposures of remimazolam.

NO.	Study	Protocols	N	C_max_ (ng/mL)			AUC_0–∞_ (ng·h/mL)		
				Obs	Pre	AFE	Obs	Pre	AFE
Artery									
1	Ia	0.007 mg/kg	3	137	174	1.27	8	8	1.08
2		0.01 mg/kg	3	269	338	1.26	16	17	1.07
3		0.04 mg/kg	3	503	881	1.75	29	43	1.51
4		0.05 mg/kg	6	960	1320	1.37	57	65	1.14
5		0.07 mg/kg	6	1270	1720	1.36	66	86	1.31
6		0.11 mg/kg	6	1710	2500	1.46	106	126	1.19
7		0.14 mg/kg	6	2690	3350	1.25	147	167	1.14
8		0.18 mg/kg	6	3740	4190	1.12	214	209	0.98
9		0.22 mg/kg	6	4250	5020	1.18	227	243	1.07
10		0.25 mg/kg	9	4170	5860	1.41	239	290	1.21
11		0.32 mg/kg	9	5790	7360	1.27	317	364	1.15
12	Ib	0.29 mg/kg + 2.16 mg/kg	8	4530	6290	1.39	2560	2800	1.09
13		0.29 mg/kg + 2.16 mg/kg	8	4530	6290	1.39	2600	2800	1.08
Vein									
14	Ia	0.32 mg/kg	9	1720	1880	1.09	273	336	1.23
15	Ib	0.29 mg/kg + 2.16 mg/kg	8	2020	1660	0.82	2971	2630	0.88
16	Ib	0.29 mg/kg + 2.16 mg/kg	8	1860	1660	0.89	2970	2630	0.88

Note: Predicted values are shown as the median of population simulations. Obs, observed; Pre, predicted.

**Table 4:** Parameter estimates of the population pharmacokinetic model of for remimazolam.

	Final Model		Bootstrap *	
	Estimates (RSE%) [Shrinkage%]	95% CI	Median	95% CI
Typical value				
CL (L/min/63 kg)	1.03 (1.9)	0.992–1.068	1.03	0.992–1.06
V1 (L/63 kg)	2.08 (2.6)	1.97–2.19	2.07	1.98–2.17
V2 (L/63 kg)	10.9 (3.8)	10.1–11.7	10.9	9.99–11.6
Q2 (L/min/63 kg)	1.49 (4.1)	1.37–1.61	1.49	1.39–1.61
V3 (L/63 kg)	19.7 (4.1)	18.1–21.3	19.5	18.1–21.1
Q3 (L/min/63 kg)	0.266 (6.1)	0.234–0.298	0.267	0.24–0.298
IIV				
ω^2^ (CL)	0.0203 (16.1) [1.5]	0.0139–0.0267	0.0199	0.0142–0.027
ω (CL): ω(V1)	0.00948 (39.7)	0.00211–0.0168	0.0098	0.00358–0.0171
ω^2^ (V1)	0.00546 (99.5) [12.4]	−0.00518–0.0161	0.0085	0.00164–0.0183
ω^2^ (V2)	0.0509 (24.4) [8.9]	0.0266–0.0752	0.0488	0.0294–0.0807
ω (V2): ω(Q2)	0.0555 (24.5)	0.0288–0.0822	0.0546	0.0318–0.0897
ω^2^ (Q2)	0.107 (18.3) [5]	0.0686–0.145	0.107	0.0706–0.15
ω^2^ (V3)	0.072 (17.5) [7]	0.0473–0.0967	0.0717	0.0488–0.0996
ω^2^ (Q3)	0.0777 (27.9) [12.2]	0.0139–0.0267	0.0767	0.0442–0.122
Error				
σ^2^ (ADD)	0.0162 (8.5) [11]	0.0135–0.0189	0.0158	0.0136–0.0177

RSE%, relative standard error; CI, confidence interval; IIV, inter-individual variability. Allometric exponents for body weight were fixed to 0.75 for clearance and 1.0 for distribution volumes. * Bootstrap results were derived from 901 successful runs out of 1000 total replicates.

**Table 5:** Target exposures for induction and maintenance phases of adult general anesthesia based on the PopPK model.

Induction Dose (mg/kg)	Maintenance Dose (mg/kg/h)	C_max_ [90% CI] (ng/mL)	C_2h_ [90% CI] (ng/mL)	AUC_0–∞_ [90% CI] (ng·h/mL)
0.3	1.0	4990 [4100, 5860]	946 [749, 1190]	2320 [1810, 2980]
	3.0	4990 [4100, 5860]	2810 [2230, 3520]	6360 [4970, 8170]

**Table 6:** Predicted PopPK to PBPK predictions (C_max_, C_2h_ and AUC_0–∞_) ratios—by age group.

Age (Years)	Weight (kg)	C_max_	C_2h_	AUC_0–∞_
Birth < 1 years	6.6	0.86	0.92	0.92
1 < 3 years	12.2	0.94	1.07	1.07
3 < 6 years	17.2	1.02	1.08	1.10
6 < 9 years	24.0	1.02	1.09	1.11
9 < 12 years	32.9	0.95	1.03	1.04
12 < 18 years	50.3	0.83	0.99	1.02
Birth > 18 years	57.0	0.75	0.94	0.98

**Table 7:** PK exposure parameters for the pediatric dosing regimen based on PopPK and PBPK model simulations.

Dose mg/kg + mg/kg/h	C_max_ (ng/mL) Median [90% CI]		C_2h_ (ng/mL) Median [90% CI]		AUC_0–∞_ (ng·h/mL) Median [90% CI]	
	PopPK	PBPK	PopPK	PBPK	PopPK	PBPK
10–30 kg						
0.3 + 1.0	4260 [3400, 5110]	4175 [3540, 4892]	735 [573, 916]	660 [532, 813]	1760 [1360, 2210]	1550 [1241, 1940]
0.3 + 4.0			2910 [2270, 3620]	2608 [2108, 3215]	6340 [4890, 7970]	5713 [4591, 7119]
30–70 kg						
0.3 + 1.0	4770 [3870, 5700]	5639 [4629, 6834]	874 [693, 1120]	881 [703, 1123]	2130 [1660, 2750]	2086 [1649, 2666]
0.3 + 3.0			2590 [2060, 3300]	2612 [2087, 3330]	5830 [4560, 7540]	5820 [4607, 7423]

## Data Availability

The datasets generated and/or analyzed during the current study are not publicly available due to patient privacy and ethical restrictions but are available from the corresponding author on reasonable request.

## Supplementary Materials

The following supporting information can be downloaded at: https://www.mdpi.com/article/10.3390/pharmaceutics18030315/s1, Supplemental Material Section S1: CES1 Ontogeny Equation; Figure S1: Ontogeny curve of CES1 fractional protein abundance in humans based on a sigmoidal maturation model; Supplemental Material Section S2: PopPK NONMEM control stream for the final model; Table S1: Demographic characteristics of analysis population; Table S2. PBPK-predicted and observed T_max_ values of Remimazolam. Table S3: Results of forward and backward stepwise covariate selection for the PopPK model. Table S4: Demographic Information of the Virtual Pediatric Population; Table S5: Demographic Information of the Virtual Pediatric Population for Dosing Recommendations; Figure S2: Average Semi-log remimazolam concentration vs. time profiles by study; Figure S3: Goodness-of-Fit Plots for the Final Model; Figure S4: Prediction-Corrected Visual Predictive Check (pcVPC) for the Final Model; Figure S5: Target exposure range for adult general anesthesia following administration of HR7056 for injection.

## Abbreviations

The following abbreviations are used in this manuscript:

ADME	Absorption, distribution, metabolism, elimination
AFE	Average fold error
ALB	Albumin
ALP	Alkaline phosphatase
ALT	Alanine aminotransferase
AST	Aspartate aminotransferase
AUC	Area under the concentration vs. time curve
BMI	Body mass index
BSA	Body surface area
BUN	Blood urea nitrogen
C2h	After dosing 2 hours concentration
CDE	Center for Drug Evaluation
CES1	Carboxylesterase 1
CI	Confidence interval
CL	Clearance
Cmax	Maximum (or peak) concentration
CR	Serum creatinine
eGFR	Estimated glomerular filtration rate
ESTs	Expressed sequence tags
FCES1	Fractional CES1 abundance in adults
FOCEI	First-order conditional estimation with interaction
GABAa	γ-aminobutyric acid A
GOFs	Goodness-of-fit plots
IIV	Inter-individual variability
IV	Intravenous
LC-MS/MS	Liquid chromatography–tandem mass spectrometry
LLOQ	Lower limit of quantification
MDRD	Modification of diet in renal disease
Obs	Observation
OFV	Objective function value
PBPK	Physiologically based pharmacokinetic
pcVPCs	Prediction-corrected visual predictive checks
PK	Pharmacokinetic
PopPK	Population pharmacokinetic
Pre	Prediction
PT	Poulin and Theil
Q	Clearance between central compartment and peripheral compartment
RR	Rodgers and Rowland
RSE	Relative standard error
RT-PCR	Reverse transcription polymerase chain reaction
SD	Standard error
TBIL	Total bilirubin
V	Volume of distribution
