# Supplementary material for: Dose Recommendation of Remimazolam Tosilate for General Anesthesia in Children and Adolescents: Synergistic Combination of PopPK and PBPK Approaches
Source: Pharmaceutics. 2026 Mar 1;18(3):315. doi: 10.3390/pharmaceutics18030315 (PMC13029219; doi:10.3390/pharmaceutics18030315)
Supplement: Supplementary file 1 [file pharmaceutics-18-00315-s001.zip › pharmaceutics-4075025-supplementary.pdf]

Supplementary Information

Dose Recommendation of Remimazolam Tosilate for General Anesthesia in Children and Adolescents: Synergistic Combination of PopPK and PBPK Approaches

Qiong-Yue Liang [1,†], Hui-Hui Hu [1,†], Nassim Djebli [1,2], Yuan-Yuan Huang [1] and Hao Jiang [1,*]

Supplemental Material S1

CES1 Ontogeny Equation

Equation terms: $Adult_{max}$, maximum average relative protein abundance; Age, age in years of the subject at the time of sample collection; Age50, age in years at which half-maximum adult protein abundance is obtained; F, fractional protein abundance in adult samples; F_{birth}, fractional protein abundance (of adult) at birth; n, exponential factor.

F_{birth}, $Adult_{max}$, Age50, and n: 0.20, 1, 1.10, and 0.56, respectively.

$$F=\left(\frac{Adult_{max}-F_{birth}}{Age50^{n}+Age^{n}}\right)\times Age^{n}+F_{birth}$$

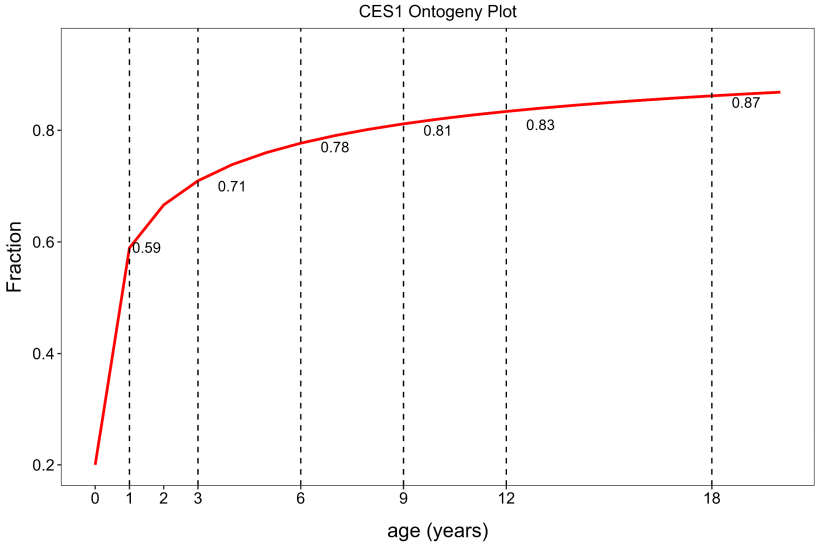

Figure S1. Ontogeny curve of CES1 fractional protein abundance in humans based on a sigmoidal maturation model.

Supplemental Material S2

PopPK NONMEM control stream for the final model:

```
$PROBLEM
;; 1. Based on: run6
;; 2. Description: 3cmt: HR7056 logDV+ADD+AS+CLV1block+V2Q2block
;; x1. Author: liangqiongyue
$INPUT C ID SUBJECTNR=DROP SUBJID=DROP STUDYID EXPDOSE DOSE GROUP
 PERIOD DATEC=DROP TIMEC=DROP TIME TALD CONC DV MDV EVID CMT AMT
 BQL RATE DUR=DROP PTAFD PTALD SEX AGE WEIGHTBL HEIGHTBL BMIBL
 BSABL ALTBL ASTBL TBILBL ALBBL ALPBL BUNBL CRBL EGFRBL DURALL ICU
 FCES1
$DATA HR7056PopPK20250627.csv IGNORE=C IGNORE=(STUDYID.GT.2)
```

```
$SUBROUTINES ADVAN11 TRANS4
$PK
      TVCL = THETA(1) * ((WEIGHTBL/62)**THETA(7))
      CL = TVCL * EXP(ETA(1))
      TVV1 = THETA(2) * ((WEIGHTBL/62)**THETA(8))
      V1 = TVV1 * EXP(ETA(2))
      TVV2 = THETA(3) * ((WEIGHTBL/62)**THETA(9))
      V2 = TVV2 * EXP(ETA(3))
      TVQ2 = THETA(4) * ((WEIGHTBL/62)**THETA(10))
      Q2 = TVQ2 * EXP(ETA(4))
      TVV3 = THETA(5) * ((WEIGHTBL/62)**THETA(11))
      V3 = TVV3 * EXP(ETA(5))
      TVQ3 = THETA(6) * ((WEIGHTBL/62)**THETA(12))
      Q3 = TVQ3 * EXP(ETA(6))
      S1 = V1/1000
$ERROR
      IPRED = LOG(0.00000001)
      IF (F.GT.0) IPRED = LOG(F)
      Y = IPRED * (1 + ERR(1)) + ERR(2)
$THETA
(0, 1.03) ;[CL (L/min/62 kg)]
(0, 2.1) ;[V1 (L/62 kg)]
(0, 11.1) ;[V2 (L/62 kg)]
(0, 1.51) ;[Q2 (L/min/62 kg)]
(0, 19.7) ;[V3 (L/62 kg)]
(0, 0.264) ;[Q3 (L/min/62 kg)]
(0.75) FIX ;[WEIGHTBL on CL]
(1) FIX ;[WEIGHTBL on V1]
(1) FIX ;[WEIGHTBL on V2]
(0.75) FIX ;[WEIGHTBL on Q2]
(1) FIX ;[WEIGHTBL on V3]
(0.75) FIX ;[WEIGHTBL on Q3]
$OMEGA BLOCK(2)
  0.0198    ; ETA1[CL]
  0.00804 0.00657    ; ETA2[V1]
$OMEGA    BLOCK(2)
  0.0393 ; ETA3[V2]
  0.01    0.0939 ; ETA4[Q2]
$OMEGA
  0.0708 ; ETA5[V3]
  0.0794 ; ETA6[Q3]
$SIGMA
  0 FIX    ; EPS[PROP]
  0.0163 ; EPS[ADD]
$ESTIMATION METHOD=1 INTER MAXEVAL=9999 PRINT=1 SIG=3
$COVARIANCE PRINT=E
$TABLE ID STUDYID GROUP PERIOD TIME DV MDV EVID PRED IPRED CWRES
CIWRES ONEHEADER NOPRINT NOAPPEND FILE=SDTAB7
$TABLE ID STUDYID GROUP PERIOD TIME CL V1 V2 V3 Q2 Q3 ETA(1) ETA(2) ETA(3)
ETA(4) ETA(5) ETA(6) NOPRINT NOAPPEND ONEHEADER FILE=PATAB7
$TABLE ID SEX STUDYID ICU NOPRINT NOAPPEND ONEHEADER FILE=CATAB7
```

```
$TABLE ID TIME AGE WEIGHTBL HEIGHTBL BMIBL BSABL ALTBL ASTBL TBILBL ALBBL ALPBL BUNBL CRBL EGFRBL DURALL DOSE FCES1 NOPRINT NOAPPEND ONEHEADER FILE=COTAB7
;; $PLOT INDIVIDUAL XAXIS=TIME
;; $PLOT GOF XAXIS=TIME
;; $PLOT CORR
; The pcVPC code: vpc -samples=1000 -auto_bin=40 -dir=pcVPC_run7 run7.mod -predcorr
```

Table S1. Demographic characteristics of analysis population.

	HR7056-Ia (N = 63)	HR7056-Ib (N = 8)	Overall (N = 71)
**Sex, n (%)**			
Female	12 (19.0%)	3 (37.5%)	15 (21.1%)
Male	51 (81.0%)	5 (62.5%)	56 (78.9%)
**Age (year)**			
Mean (SD)	28.9(6.4)	26.4(4.9)	28.6(6.3)
Median [Min, Max]	28.0[18.0, 51.0]	25.5[21.0, 34.0]	27.0[18.0, 51.0]
**Weight (kg)**			
Mean (SD)	64.0(7.3)	58.7(5.8)	63.4(7.3)
Median [Min, Max]	63.4[52.8, 83.8]	57.8[50.2, 68.2]	62.8[50.2, 83.8]
**Height (cm)**			
Mean (SD)	168.3(6.4)	167.6(6.0)	168.2(6.4)
Median [Min, Max]	168.0[156.0, 184.0]	168.0[155.0, 174.0]	168.0[155.0, 184.0]
**BMI (kg/m^2)**			
Mean (SD)	22.6(1.8)	20.9(1.1)	22.4(1.8)
Median [Min, Max]	22.6[18.4, 25.7]	21.0[19.3, 22.8]	22.3[18.4, 25.7]
**ALT (U/L)**			
Mean (SD)	15.3(6.0)	9.2(4.4)	14.6(6.2)
Median [Min, Max]	14.0[6.0, 33.0]	8.0[5.0, 18.0]	13.0[5.0, 33.0]
**AST (U/L)**			
Mean (SD)	19.2(3.8)	16.1(2.7)	18.8(3.8)
Median [Min, Max]	19.0[12.0, 32.0]	16.5[11.0, 20.0]	19.0[11.0, 32.0]
**TBIL (umol/L)**			
Mean (SD)	11.9(5.0)	14.1(3.5)	12.2(4.9)
Median [Min, Max]	10.7[5.3, 26.2]	13.9[9.9, 19.2]	11.2[5.3, 26.2]
**CR (umol/L)**			
Mean (SD)	70.4(13.0)	69.9(13.6)	70.4(13.0)
Median [Min, Max]	72.0[46.0, 100.0]	70.0[49.0, 91.0]	72.0[46.0, 100.0]
**EGFR (mL/min)**			
Mean (SD)	53.7(20.3)	59.7(22.0)	54.3(20.4)
Median [Min, Max]	47.5[27.9, 107.7]	50.4[36.9, 96.2]	48.3[27.9, 107.7]
**BUN (mmol/L)**			
Mean (SD)	4.7(1.3)	3.9(1.5)	4.6(1.4)
Median [Min, Max]	4.6[2.3, 8.4]	3.4[1.9, 6.1]	4.5[1.9, 8.4]
**FCES1**			
Mean (SD)	0.888(0.011)	0.883(0.010)	0.887(0.011)
Median [Min, Max]	0.888[0.862, 0.916]	0.882[0.871, 0.898]	0.886[0.862, 0.916]

Table S2. PBPK predicted and observed T_{max} values of Remimazolam.

NO.	Study	Protocols	N	T_{max} (min)		
				**Obs**	**Pre**	**AFE**
**Artery**						
1	Ia	0.007 mg/kg	3	1.00	1.05	**1.05**
2		0.01 mg/kg	3	1.00	1.05	**1.05**
3		0.04 mg/kg	3	1.00	1.05	**1.05**
4		0.05 mg/kg	6	1.00	1.05	**1.05**
5		0.07 mg/kg	6	1.00	1.05	**1.05**
6		0.11 mg/kg	6	1.00	1.05	**1.05**
7		0.14 mg/kg	6	1.00	1.05	**1.05**
8		0.18 mg/kg	6	1.00	1.05	**1.05**
9		0.22 mg/kg	6	1.00	1.00	**1.00**
10		0.25 mg/kg	9	1.00	1.00	**1.00**
11		0.32 mg/kg	9	1.00	1.05	**1.05**
12	Ib	0.29 mg/kg + 2.16 mg/kg	8	1.00	1.05	**1.05**
13		0.29 mg/kg + 2.16 mg/kg	8	1.00	1.05	**1.05**
**Vein**						
14	Ia	0.32 mg/kg	9	1.00	1.55	1.55
15	Ib	0.29 mg/kg + 2.16 mg/kg	8	2.00	1.65	0.83
16	Ib	0.29 mg/kg + 2.16 mg/kg	8	2.00	1.65	0.83

Note: Predicted values were shown as the median of population simulations. Obs, Observed; Pre, Predicted.

Table S3. Results of forward and backward stepwise covariate selection for the PopPK model.

Step	Covariate screening	OFV	Δ OFV	p-value	Comments
1	weight (AS)	-3632.98309			Base model
Forward inclusion					
2	V2 + sex	-3643.72524	-10.74215	0.001047	
3	V2 + sex / CL+BUN	-3651.96592	-8.24068	0.004096	
4	V2 + sex / CL+BUN / V1+ALB	-3656.57770	-4.61179	0.031753	Full model
Backward elimination					
5	V2 + sex / CL+BUN	-3651.96591	4.61179	0.031753	
6	V2 + sex	-3643.72524	8.24068	0.004096	
7	weight (AS)	-3632.98310	10.74214	0.001047	Final model

Note: AS, allometric scaling based on body weight; ΔOFV, the change of OFV. Covariates were included during forward selection if the decrease in OFV exceeded 3.84 ($p < 0.05$, df = 1) and retained in the final model during backward elimination if the increase in OFV was less than 10.83 ($p < 0.001$, df = 1).

Covariate–parameter relationships evaluated: CL=sex, age, height, ALT, AST, TBIL, ALB, ALP, BUN, eGFR, FCES1; Q2=sex, age, height, ALT, AST, TBIL, ALB, ALP, BUN, eGFR; Q3=sex, age, height, ALT, AST, TBIL, ALB, ALP, BUN, eGFR; V1=sex, age, height, ALB, ALP; V2= sex, age, height, ALB, ALP; V3= sex, age, height, ALB, ALP.

Table S4. Demographic Information of the Virtual Pediatric Population.

Cohorts	Age (years) Median [min, max]	Weight (kg) Median [min, max]	Height (cm) Median [min, max]	BMI (kg/m2) Median [min, max]
1	0.5 [0.0–1.0]	6.6 [3.1–11.3]	67.6 [49.2–84.3]	14.8 [11.2–20.4]
2	1.8 [1.0–3.0]	12.2 [9.0–17.7]	88.4 [74.1–102.1]	15.8 [13.1–22.4]
3	4.4 [3.1–5.9]	17.2 [12.9–25.7]	107 [93.0–123.4]	15.1 [12.9–19.9]
4	7.6 [6.0–9.0]	24.0 [16.1–49.0]	124 [102–141.9]	15.5 [12.4–27.9]
5	10.4 [9.1–12.0]	32.9 [25.0–48.7]	139 [123–159.1]	16.7 [12.7–23.9]
6	15.4 [12.1–18.0]	50.3 [33.9–69.9]	159 [145–181.0]	19.8 [15.7–27.7]
7	38.7 [18.3–58.4]	57.0 [41.6–82.1]	163 [138–182]	21.0 [17.4–32.3]

Table S5. Demographic Information of the Virtual Pediatric Population for Dosing Recommendations.

Cohorts	Weight (kg) Median [min, max]	Height (cm) Median [min, max]	BMI (kg/m2) Median [min, max]
≤ 30 kg	20.3 [11.1–30]	114.7 [85.7–140]	15.3 [12.5–22.1]
> 30 kg	46.3 [30.1–69.8]	154 [121–187]	19.1 [14.3–33.0]

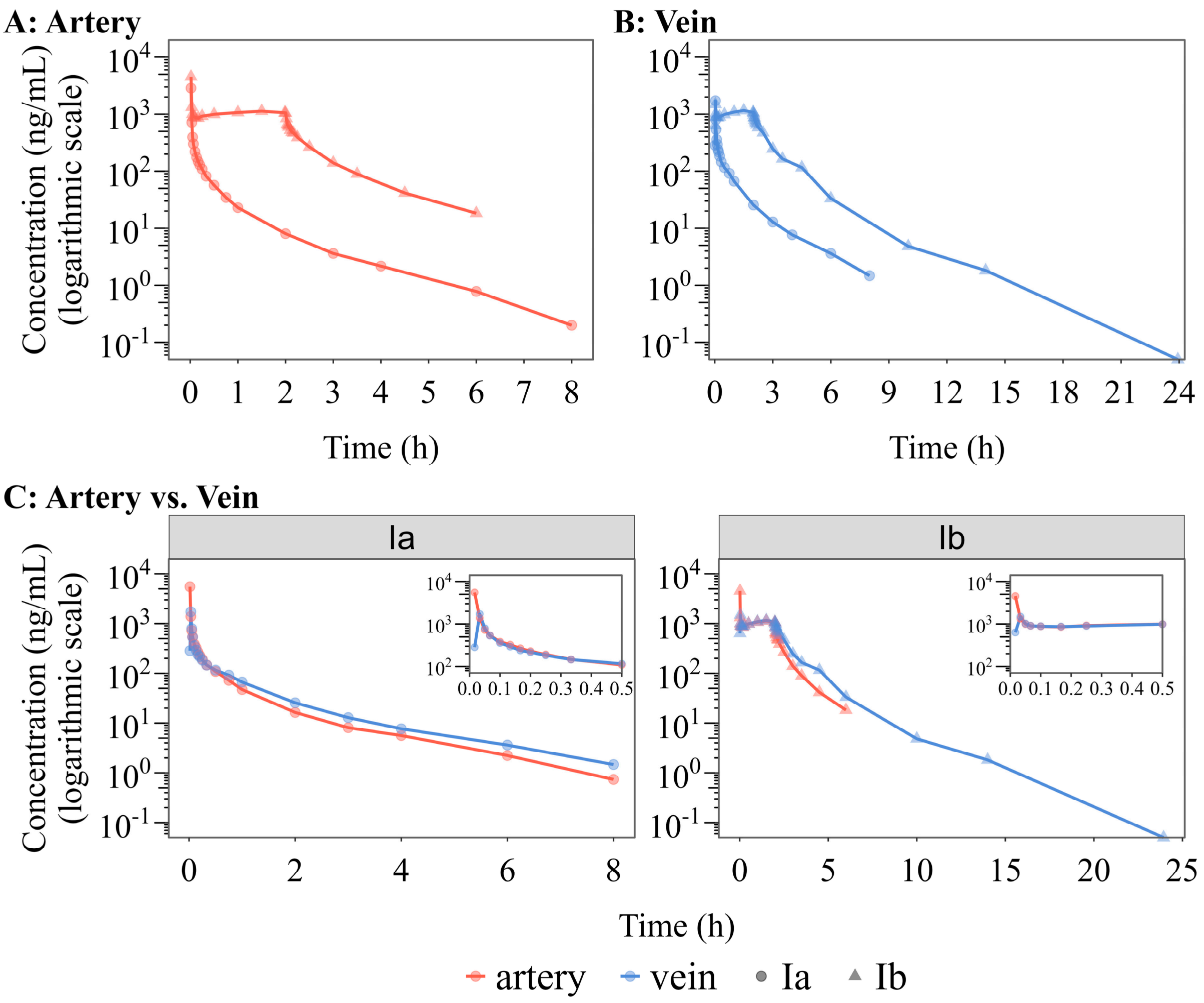

Figure S2. Average Semi-log remimazolam concentration vs. time profiles by study. (A) Mean Arterial Concentration-Time Profiles. (B) Mean Intravenous Concentration-Time Profiles. (C) Comparative Mean Concentration-Time Profiles.

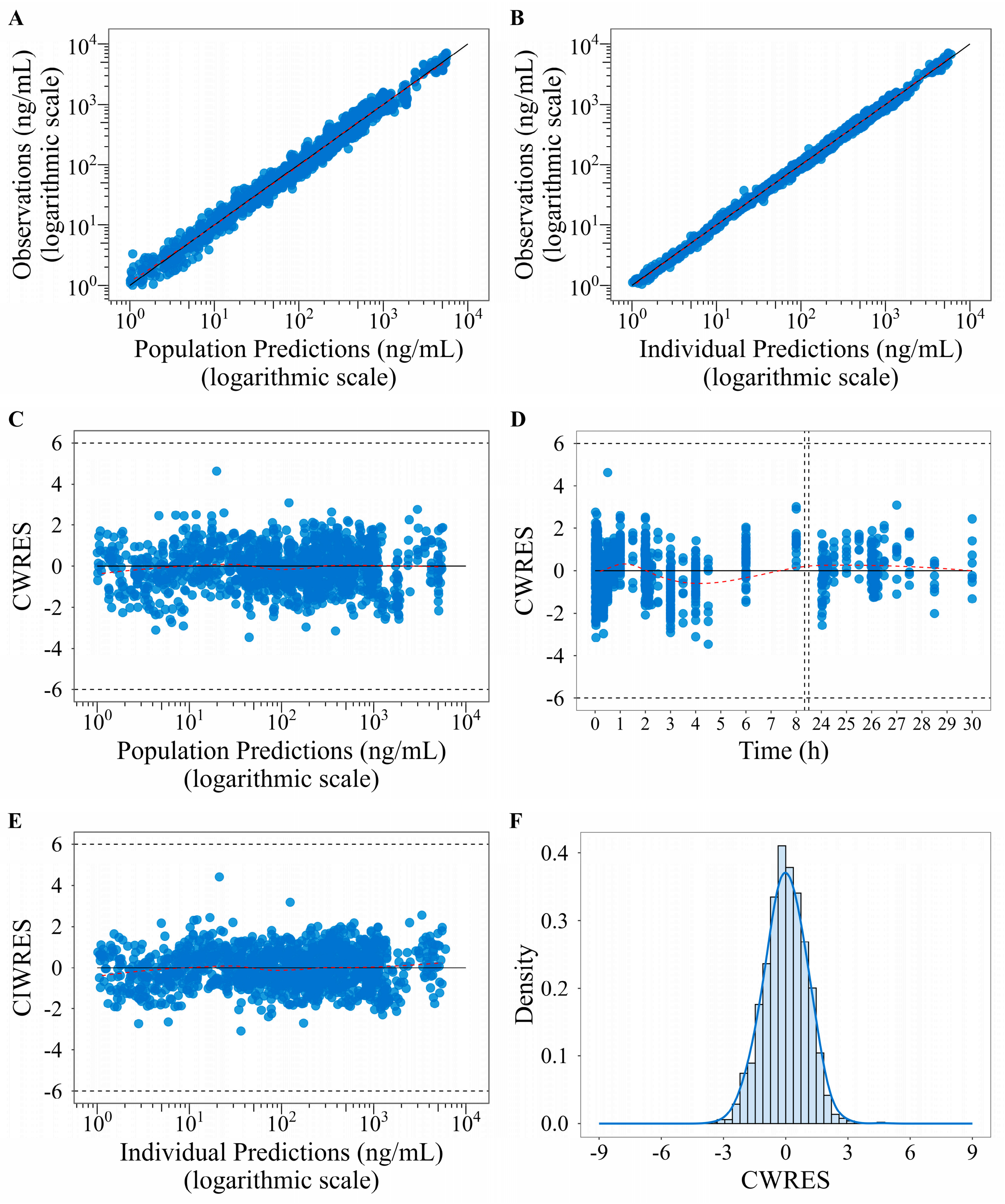

Figure S3. Goodness-of-Fit Plots for the Final Model. Observed, population-predicted, and individual-predicted values are plotted on log-transformed axes. A: Population-predicted values vs. observed values; B: Individual-predicted values vs. observed values; C: Conditional weighted residuals vs. population-predicted values; D: Conditional weighted residuals vs. time; E: Individual conditional weighted residuals vs. individual-predicted values; F: Histogram of conditional weighted residuals. The black solid line represents the line of identity, and the red line represents the trend line.

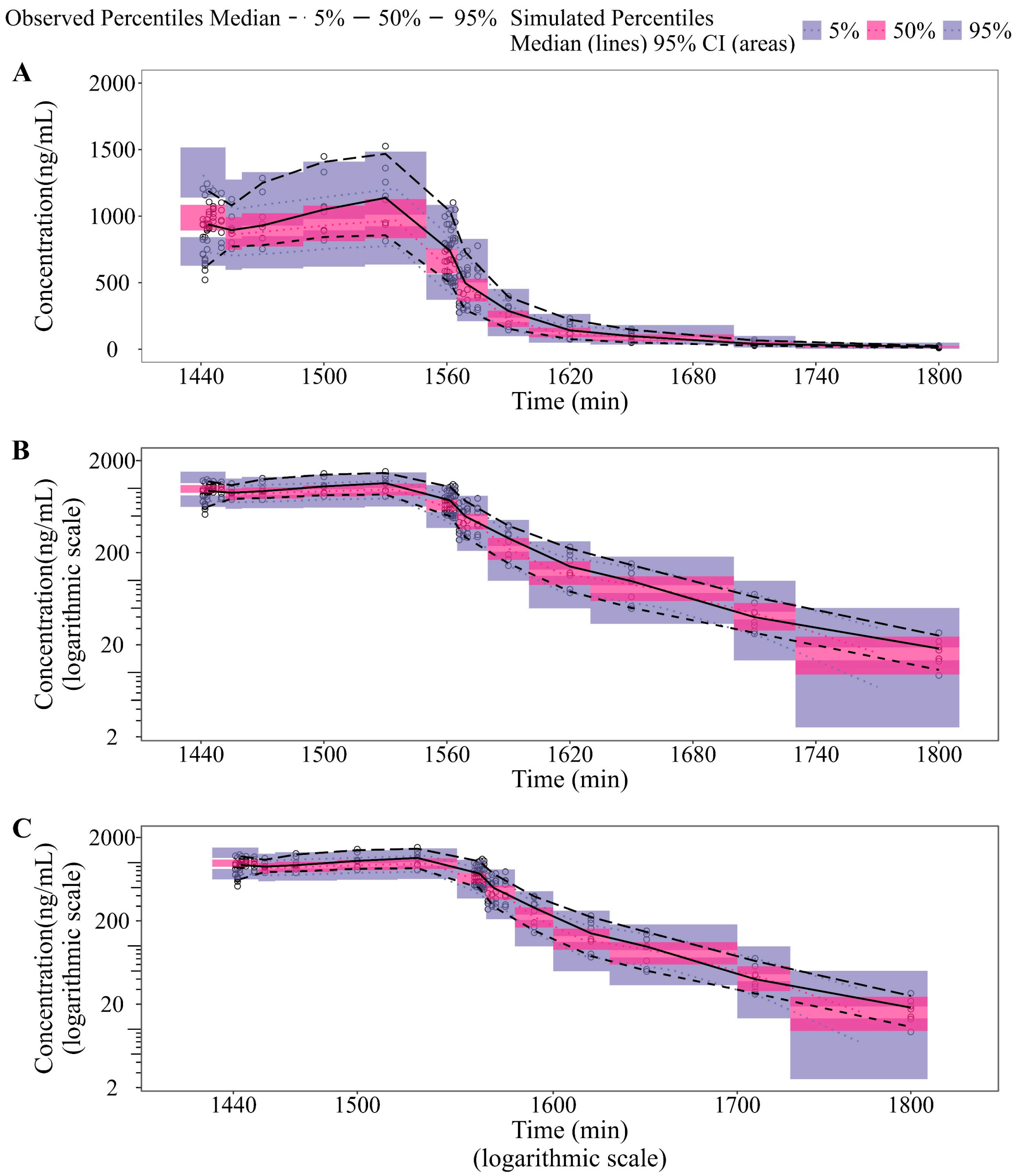

Figure S4. Prediction-Corrected Visual Predictive Check (pcVPC) for the Final Model. The 95th (black long-dashed line), 50th (black solid line), and 5th (black short-dashed line) percentiles of the observed data are shown. The 95th (black dotted line), 50th (red dotted line), and 5th (black dotted line) percentiles of the simulated data are also shown. The shaded area represents the 95% confidence interval for the corresponding percentiles of the simulated data. Open black circles represent observed values. A: pcVPC plot on a linear scale; B: pcVPC plot with a semi-logarithmic Y-axis; C: pcVPC plot with a log-log scale.

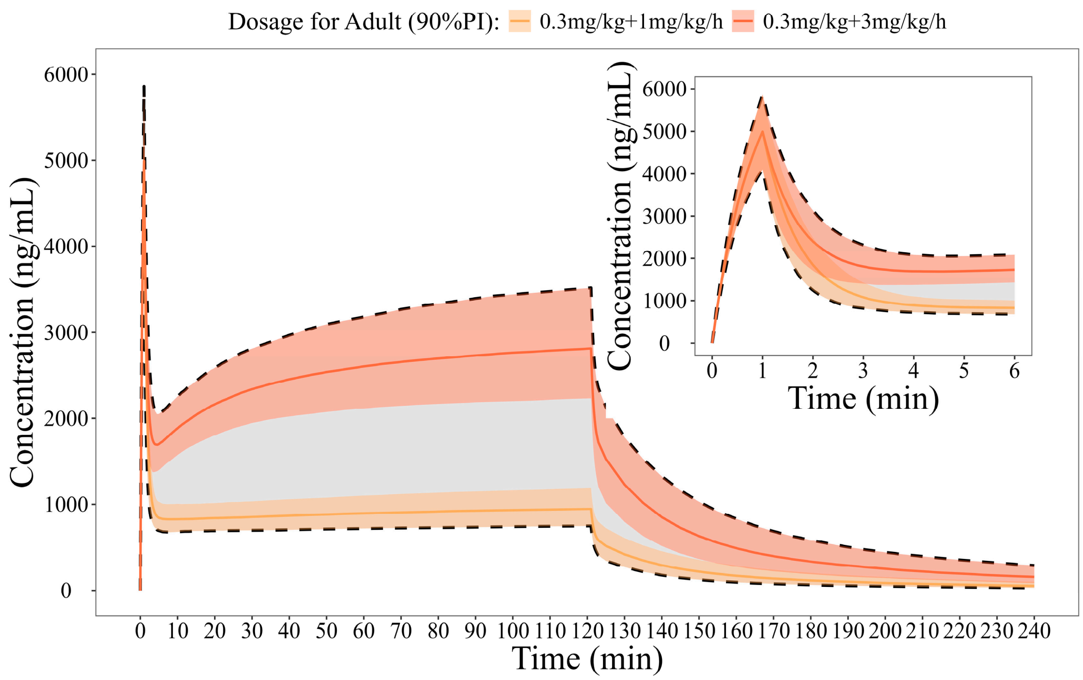

Figure S5. Target exposure range for adult general anaesthesia following administration of HR7056 for injection. The black dashed area represents the target exposure range for adult general anaesthesia, defined as the 5th percentile of the regimen with an induction dose of 0.3 mg/kg followed by a maintenance infusion of 1.0 mg/kg/h (lower bound) and the 95th percentile of the regimen with 0.3 mg/kg induction and 3.0 mg/kg/h maintenance (upper bound). Coloured solid lines and shaded areas indicate the median and 5th–95th percentile ranges for the 1.0 mg/kg/h and 3.0 mg/kg/h maintenance regimens, respectively.
